# Achieving sustainability in health information systems: a field tested measure of country ownership

**DOI:** 10.1186/s12992-017-0258-0

**Published:** 2017-06-24

**Authors:** Stephanie Watson-Grant, Khou Xiong, James C Thomas

**Affiliations:** 1 MEASURE Evaluation project, Carolina Population Center, University of North Carolina, Chapel Hill, NC USA; 2 Department of Epidemiology, Gillings School of Global Public Health, University of North Carolina, Chapel Hill, NC USA

**Keywords:** Health information system, Sustainability, Sustainable development goals, Country ownership

## Abstract

**Background:**

A country will trust, value, and use, its health information system (HIS) to the extent it has had a role in its creation and maintenance. A sense of ownership contributes in turn to the long-term sustainability of the HIS, and thus the country’s ability to monitor and evaluate population health and health services. To facilitate progress toward greater ownership, we developed and tested a tool to measure the country’s ownership of its monitoring and evaluation (M&E) system.

**Methods:**

Through a systematic review of the literature, we identified four dimensions of country ownership of an M&E system: partnership, commitment and responsibility, capacity, and accountability. We identified relevant indicators of the dimensions already in use in other tools used to assess M&E systems. We tested the data collection tool with 95 stakeholders of the Tanzanian HIS for HIV/AIDS control.

**Results:**

We identified 56 items that addressed elements of the four dimensions. The respondents found our tool for assessing country ownership of an HIS to be clear and relevant, leading to the identification of important issues to be discussed. For example, all stakeholder groups affirmed that the Tanzanian Commission for AIDS is “playing a leadership role in addressing HIV through collaborative partnerships and work across borders to achieve greater impact.” While many respondents disagreed with the statement, “There is an adequate number of government monitoring and evaluation posts at the sub-national level.”

**Conclusions:**

Stakeholders found the M&E country ownership tool to address relevant questions clearly. It enabled them to identify successes and challenges within four dimensions of country ownership. It thus holds the potential to lead to an agenda for strengthening country ownership. If implemented every few years, the tool can provide a means of monitoring progress through a set of standardized indicators. As country ownership of M&E increases, so will the long-term sustainability of the HIS.

## Background

To achieve and maintain healthy populations, countries need data they can trust for monitoring progress and evaluating programs. Their trust in the data will rest in part on their familiarity with the health information system (HIS) that enables monitoring and evaluation (M&E), and their role in its creation and maintenance. Their roles and their trust are often summarized in the term country ownership. To ensure it is achieved, can country ownership itself be measured and monitored?

The 2030 Agenda for Sustainable Development asserts that “every State has, and shall freely exercise, full permanent sovereignty over all its wealth, natural resources and economic activity” [1]. This statement points indirectly to a tension inherent to international development, in which wealthy external donors provide resources that low- and middle-income countries (LMICs) lack. In so doing, the donors hold the potential to assert their own agendas over the LMICs. Respecting the sovereignty of the recipient country in this context is referred to as “country ownership.” The concept has been central in international discussions about sustainable development in Paris [2], Accra [3], and Busan [4]. The primary principle is that when country stakeholders have not taken part in the planning or implementation of a strategy, they have little motivation to assume it after the donors have ended their involvement.

The US Government, as articulated in the Global Health Initiative, sees country ownership as a key principle of the collective investment in all areas of health in developing countries [2]. Specifically, it “encourages sustainable country-owned programs when it promotes direct financing by recipient countries for priority interventions such as malaria and family planning commodities. Ultimately, a well-coordinated, country-led health response enhances efficient use of resources and contributes to long-term sustainability of heath programming.”

An underlying tenet of the SDGs is that “what gets measured, gets done.” The 230 indicators provide quantified goals and a means of monitoring progress towards them. However, none of them explicitly addresses country ownership. Nor is there a measure of country ownership available elsewhere. Yet, as with the SDGs, measurement holds the potential to facilitate progress. We sought to develop a measure of country ownership of the M&E system embedded in the country HIS - one that is detailed enough to cover its multiple dimensions, and accessible enough to be readily understood, trusted, and implemented by country personnel. To achieve this, through the MEASURE Evaluation project we conducted a literature review, constructed a tool, and tested it in Tanzania. Our aims in this paper are to describe the tool and its development, and how it was regarded in the first instance of implementation.

## Methods

### Identifying dimensions

To identify the dimensions of country ownership we reviewed both peer-reviewed and non-peer-reviewed (“grey”) literature. We searched for peer-reviewed articles published since June 2005 with the University of North Carolina’s e-research tools, which draw from more than 350 English language databases, including PubMed, Web of Science, JSTOR Arts & Sciences Collection, and Wiley InterScience Journals. We searched the grey literature through a Google search on the term “country ownership.” This search yielded 79 articles and reports. This number was reduced to 30 after eliminating duplicates, announcements of forums where country ownership would be discussed, and articles where country ownership was mentioned but not defined or described.

We used an iterative template analysis approach to identify key themes [5]. An initial set of 21 code words was constructed from a word frequency analysis of the 30 articles and reports. To group them into parent codes, we read the articles for clustering of the terms. The parent codes were refined upon rereading of the 30 articles and reports.

### Constructing a data collection tool

In view of critiques and warnings about the proliferation of indicators, e.g., by Boerma [6] and the World Health Organization [7], we sought components of existing tools that could provide useful information on the dimensions of country ownership. We searched the websites of leading international health organizations, government relief agencies, and organizations known for one or more of the country ownership dimensions for potential tools and identified 19 tools. After reviewing each, eight were discarded because they had no actual measurement component or were not relevant to the issues of country ownership. We incorporated the remaining 11 into an Excel-based data collection tool [8–18].

We used a five-point Likert-type scale of “strongly agree” to “strongly disagree” for responses to the questions or statements. The middle or neutral answer of the five was worded as “no answer” in order to cover instances in which a respondent lacked sufficient information to respond.

### Data collection and analysis

We tested the data collection tool in June 2014 among stakeholders aiming to use the Tanzanian health information system (HIS) for HIV/AIDS control. Stakeholder categories were selected according to their roles and functions identified in the national multi-sectoral HIV monitoring and evaluation system, which is coordinated by the national Tanzania Commission for AIDS (TACAIDS).

Over a 3-week period, four MEASURE Evaluation researchers conducted face-to-face interviews lasting 60–90 min with 95 respondents from six stakeholder groups: TACAIDS; the Tanzania Ministry of Health; international non-government organizations; representatives from the country’s 29 regions; development partners; and government ministries, departments, and agencies.

The number of respondents answering at one level of the comments for example level 2, or “agree”) was expressed as a percentage of all responses. The percentages, summing to 100%, were then visualized with a horizontal bar, with a different color representing each response level.

Synopses of qualitative comments volunteered by respondents during the interviews were recorded in the tool by the interviewers. The responses were mainly in Likert scale with additional comments noted by the interviewers. Therefore, the synopsis of comments was grouped by country ownership dimensions and elements. In addition, all respondents were invited to a meeting to discuss and interpret study results with the researchers.

Ethics review and approval for data analysis and publication of results were obtained from the University of North Carolina public health and nursing Institutional Review Board (IRB). The study entails no personal or private information and was declared not human subjects research.

## Results

### Concept dimensions

In our review of the literature, the final four parent codes from the template analysis, herein referred to as the dimensions of country ownership, were (1) partnership, (2) commitment and responsibility, (3) capacity, and (4) accountability (Table 1).

**Table 1:** Parent codes and sub-codes from the template analysis of 30 articles and reports describing country ownership

Parent code	Sub-codes
Partnership	Foreign assistance, leadership, rights, influence, power, legitimacy, respect
Commitment and Responsibility	Good governance, development, partnership, commitment, planning, responsibility, alignment, efficiency
Capacity	Capacity, capacity building, technical assistance, sustainability
Accountability	Accountability, standards

The concept of partnership incorporates the power of donors and the rights of recipients, and the parameters for engagement in this relationship [3, 19–24]. Twelve statements addressed partnership. Two examples were “The TACAIDS is empowered to take action to adjust program implementation” and “International partners made changes in their programming strategies to effectively support the HIV program.”

Commitment to and responsibility for an HIS by donors and the recipient country was addressed in 13 statements about responding to any failed outcomes of an HIS system funded by donors [22, 25]; ensuring the necessary leadership, governance, and operational structures should be in place [19]; and donor commitment to the processes being undertaken and share in the responsibility for failure or success [26, 27]. An example question was “The AIDS authority has a well-defined strategic plan that sets clear national priorities that are linked to functioning systems and respond to unique local conditions.”

Recipient capacities were addressed in 19 statements about the necessary individual, organizational, and systemic capacities to maintain an HIS [19, 28, 29] Where the capacity is lacking, donors should provide technical assistance to develop a capacity building plan and activities [27, 30, 31]. An example statement was “There is an adequate number of government M&E posts at the sub-national level.”

Relevant types of accountability included recipients to donors, recipients to citizens, and donors to recipients and their own citizens [3, 26, 32, 33]. The achievement of accountability is predicated on high quality, relevant data that are shared with local, national and international stakeholders [3, 27, 34]. This dimension was addressed with 12 items. One example was “M&E staff who submit reports consistently get feedback.”

The tool is available free of charge on the project website.

### Field testing in Tanzania

**Fig. 1:**
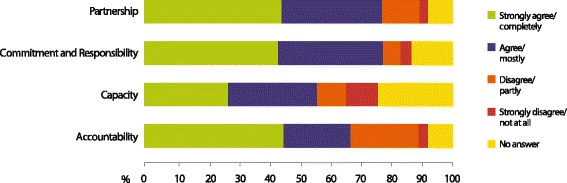
Summary of responses to the 2014 Tanzania implementation of the tool measuring country ownership of the monitoring and evaluation system administered to 95 respondents in 29 stakeholder agencies and organizations

We tested the tool through interviews conducted with 95 participants, representing 29 stakeholder agencies or organizations. All those invited to participate in the interview agreed to do so. Our primary interest was not in the state of country ownership of the Tanzania’s HIS, but in the value of the instrument for collecting, quantifying, and synthesizing information to inform and facilitate discussions among the stakeholders. We present a summary of the responses in Fig. 1, below.

None of the respondents terminated the interview before it was finished; and none mentioned that any of the questions was unclear. In some instances, respondents lacked the information needed to answer the question (see “no answer” responses in Fig. 1), but this in itself provided valuable data about the accessibility of information that should be made available to all HIS stakeholders. For example, there were many “no answer” responses to the statement “The AIDS authority’s annual implementation plans have elements that can be mapped directly to the elements of the HIV strategic plan.” Likewise, in qualitative statements during the interview, several respondents wondered whether the TACAIDS had any operational plans at all. Many believed there was an operational planning process, but no document serving as a TACAIDS operational plan.

The instrument also directly identified a number of strengths and weaknesses in Tanzania’s dimensions of country ownership. For example, with regard to capacity, two statements that received positive responses from all stakeholder groups were (1) “The AIDS authority leadership uses structured processes for planning and managing change,” and (2) “The AIDS authority is playing a leadership role in addressing HIV through collaborative partnerships and work across borders to achieve greater impact.” In contrast, many respondents disagreed with the statement “There is an adequate number of government monitoring and evaluation posts at the sub-national level.” Findings such as these can point stakeholders to the elements of country ownership or sustainability most needing their attention.

Patterns in types of responses by various stakeholder groups suggested different perspectives, or perhaps biases, in the responses. We noted, for example, that respondents representing the TACAIDS agreed with statements more often than other stakeholder groups; and respondents from the MOH were more likely than others to disagree with the statements. The specificity of the statements in the instrument would facilitate productive discussion of these different perspectives, either enabling the two to reach common ground, or point to additional information needed.

### Stakeholder discussions about the instrument

In the meeting following implementation of the tool, a number of the questionnaire items stimulated discussion among the respondents. One such item was the statement, “M&E tasks that are usually the responsibility of government can be fulfilled without external M&E technical support.” This led the respondents to explore together the sources of support for data collection, flow, and analysis in the country, and whether they were adequate for country needs. The statement, “The AIDS authority has well established systems for human resource planning and management of human resource resources, and procedures to support current and anticipated levels of M&E of HIV,” led several respondents to consider the transition from external donor to country resources, and long-term planning processes. They mentioned in particular the Tanzanian AIDS Trust Fund that was started but had yet to achieve much momentum.

When asked about the utility of the questionnaire items, some noted the value of the “no answer” response option, revealing their knowledge gaps, and enabling them to reflect on their engagement with HIS processes. The most common comment was that the tool made them think of things they should know but didn’t. The data visualization was readily understood. One respondent noted that it gave a very ‘satisfying summary’ that tied the tool and the concept together. Some questioned whether it was reasonable to expect certain respondents (e.g., sub-national stakeholders) to know the details of the partnership process.

## Discussion

We sought to develop a measure of country ownership of the M&E system that is detailed enough to cover its multiple dimensions; and accessible enough to be readily understood, trusted, and implemented by country personnel. The respondents in Tanzania found our tool to be clear and relevant, leading to the identification of important issues to be discussed. The perceived clarity would enable ease of future implementation by country personnel. The perceived relevance suggests we captured the multiple dimensions. Furthermore, the tool relevance would facilitate trust in the results. The presentation of survey results to a gathering of the stakeholders led to fruitful discussions that, in time, could result in a shared agenda. Further evidence of utility would eventually be priorities and actions emerging from the stakeholder discussions to enhance country ownership.

The four dimensions of country ownership we identified were similar to those simultaneously and independently identified in a USAID commissioned white paper [2]. The paper identified: (1) political ownership and stewardship, (2) institutional and community ownership, (3) capabilities, and (4) mutual accountability, including finance. The white paper does not describe the methods by which their four dimensions were identified. A footnote in the white paper noted they were informed by the 2005 Paris Declaration on Aid Effectiveness [3], the 2008 Accra Agenda for Action [3], a 2010 USAID publication on country ownership in the context of Rwanda [13], and interviews conducted by McKinsey and Company with leaders of various US agencies, the United Nations Joint Programme on HIV/AIDS (UNAIDS), and country stakeholders in Botswana and South Africa [14].

The first two categories in the white paper (political ownership and stewardship, and institutional and community ownership), were worded differently than two of our dimensions (commitment and responsibility, and partnership), but elements within theirs were similar to statements characterizing ours. For example, the authors said a characteristic of political ownership and stewardship is “National plans are aligned to national priorities to achieve planned targets and results, with full costing estimates and plans incorporated.” Two of the indicators of our dimension of commitment and responsibility are “the AIDS Authority’s annual implementation plan has elements that can be mapped directly elements of the HIV strategic plan” and “the national implementation plan defines technical and/or cost sharing responsibilities for development partners and the government.” The last two categories delineated in the white paper (capabilities and mutual accountability) are nearly identical to two we identified (capacity and accountability). The two sets of dimensions are somewhat different, yet strikingly similar. The independent replication serves to increase confidence in the construct validity, and thus the utility of our measurement tool.

In the meeting to discuss study findings, respondents reported that they found our instrument to be clear and that it addressed relevant issues. The respondent’s answers identified some aspects of country ownership that were well under way, and others that needed attention. For example, the fact that they were not aware of the relevant document, the *Tanzania National Multisectoral HIV and AIDS Monitoring and Evaluation Plan 2010–2012* [35], is an indication of needed improvement in communication and coordination.

The respondents also pointed to room for improvement in the instrument. Some felt the statements about accountability missed some important aspects of the issue and that could be added in future versions. And some wondered whether local-level stakeholders should be expected to know what is going on at the national level. Implementation of the instrument in other countries would likely reveal whether these concerns are broadly shared, and if so, how the statements in the instrument should be reworded or the perhaps shaped according to particular stakeholder groups.

MEASURE Evaluation constructed the instrument with an eye to country ownership, but it also captured many elements of the broader issue of sustainability in that long-term maintenance of and investment in an HIS depends heavily on a sense of relevance, utility and ownership. Even so, there are important elements of sustainability not addressed by this instrument. For example, the Ebola epidemic in West Africa highlights the importance of a system being able to withstand a substantial shock—i.e., demonstrate resilience, which contributes to sustainability—yet this instrument does not provide information on the ability of the HIS to withstand such a shock.

Restricting the instrument to the elements of country ownership of an HIS that address HIV and AIDS control allowed for precise definitions within each dimension. In turn, the clarity of what was being addressed enabled reliable responses and facilitated discussion of the summarized results. When seeking to understand country ownership in other aspects of the health system, say healthcare delivery—or in another sector, such as education—the statements would need to be customized to the situation. We also suggest that those attempting measurement of country ownership outside of an HIS reassess the relevant dimensions before compiling a list of indicators.

Although we demonstrated that measurement of country ownership facilitates discussions which, in turn, can lead to an agenda for improvements, we do not believe that scores generated through use of the tool present a fully objective and replicable representation of country ownership. As such, we do not suggest that the instrument be used to rank one country against another. Repeat implementation within a given country may show signs of change over time, but one should interpret with caution any quantitative changes, such as the percent of respondents strongly agreeing with a particular statement. We do suggest, however, that any changes be discussed by a group of stakeholders to see what they can discern about the reasons for the change.

## Conclusions

Country ownership includes echoes of historical relationships between countries, fluctuations in funding levels and funding mechanisms, changing national economies, changes in disease patterns, and much more. The complexity and dynamic nature of these elements might be enough to discourage attempts to measure them. However, we identified a set of questions that allow stakeholders to collect clear and useful information on four key elements of a country’s ownership of the HIV M&E system. With appropriate caution given to interpretation of the quantitative values, the results can help stakeholders identify the elements needing attention, facilitate discussion about reasons for any lack of progress, and move towards a shared agenda for improvement. The instrument provides a framework and a common language for discussions. Eventually, the cumulative experiences of several countries with the instrument will inform whether and how improvements can be made, and how they can result in a sustainable HIS.

## Abbreviations

AIDS: Acquired immunodeficiency syndrome
HIS: Health information system
M&E: Monitoring and evaluation
SDG: Sustainable development goal
TACAIDS: Tanzania Commission for AIDS
